# The Effect of a Host on the Primary Metabolic Profiling of Cuscuta Campestris’ Main Organs, Haustoria, Stem and Flower

**DOI:** 10.3390/plants10102098

**Published:** 2021-10-03

**Authors:** Krishna Kumar, Rachel Amir

**Affiliations:** 1 Migal—Galilee Technology Center, P.O. Box 831, Kiryat Shmona 1101600, Israel; krishnasahni16@gmail.com; 2 Tel-Hai College, Kfar Giladi 1220800, Israel

**Keywords:** GC-MS analysis, holoparasitic plant, metabolic changes, parasitic organs

## Abstract

*Cuscuta campestris* (dodder) is a stem holoparasitic plant without leaves or roots that parasitizes various types of host plants and causes damage to certain crops worldwide. This study aimed at gaining more knowledge about the effect of the hosts on the parasite’s levels of primary metabolites. To this end, metabolic profiling analyses were performed on the parasite’s three main organs, haustoria, stem and flowers, which developed on three hosts, *Heliotropium hirsutissimum*, *Polygonum equisetiforme* and *Amaranthus viridis*. The results showed significant differences in the metabolic profiles of C. campestris that developed on the different hosts, suggesting that the parasites rely highly on the host’s metabolites. However, changes in the metabolites’ contents between the organs that developed on the same host suggest that the parasite can also self-regulate its metabolites. Flowers, for example, have significantly higher levels of most of the amino acids and sugar acids, while haustoria and stem have higher levels of several sugars and polyols. Determination of total soluble proteins and phenolic compounds showed that the same pattern is detected in the organs unrelated to the hosts. This study contributes to our knowledge about the metabolic behavior of this parasite.

## 1. Introduction

*Cuscuta campestris* L., also known as dodder, is one of more than 180 species that belong to the Convolvulaceae family. C. campestris is a stem holoparasitic plant, an extensive climber having filiform stems or vines with twining slender stems lacking true roots and leaves. It has a reduced or absent photosynthesis apparatus, therefore, its development and growth rely on autotropic host plants for at least carbohydrates [1,2,3]. After proper attachment to the host’s vascular system (xylem and phloem) through the haustoria [2], the parasite functions as an active sink, redirecting solutes away from autotrophic sink tissues. Due to this parasitism, C. campestris is considered to be among the most destructive agricultural weeds, significantly reducing the yield and quality of the crops’ products [3,4,5,6]. It attacks many broad-leaf plants, the most sensitive of which include alfalfa, carrot, tomato, sugar beet, onion, potato and several ornamental plants [3]. C. campestris is native to central North America, but in recent decades, it has spread around the world through seed imports, a process that is still ongoing, and thus has become one of the most problematic weeds worldwide [7].

Despite knowledge accumulated on the parasite’s lifecycle, mode of action and agricultural damage, our knowledge about the metabolites absorbed by the parasite and how the host affects its primary metabolic profiling is still mostly unknown [6]. Moreover, there is debate in the literature as to whether most of the metabolites found in holoparasites such as *C. campestris* are taken from the host, or whether the parasite produces most of its metabolites on its own, relying primarily on the sugars transported from its hosts. Some studies suggest that the parasite obtains all its necessary compounds from its hosts, including photosynthetic metabolite, nitrogen, macro/micro minerals, water, phytohormones and other primary and secondary metabolites, as well as RNA and proteins (e.g., [1,2,8,9]). However, it was recently found in the C. campestris genome, that this parasite has genes that can function in the synthesis of primary metabolites, such as all fatty acids and amino acids, as well as co-enzymes and vitamins [10,11]. In addition, several reports suggest that holoparasitic plants can self-regulate their own metabolites since their metabolic profiles differ significantly from their hosts [8,12,13,14]. Still, numerous questions remain unanswered regarding the independence of the parasite from the metabolisms of its host [6].

Metabolomics has proven to be a powerful method for identifying metabolites whose levels are altered during development and growth conditions, as well as in response to stress [15,16]. Such an analysis could also be proposed for putative biochemical pathways and provide more data on the metabolism and physiological processes [15]. Metabolomics techniques have rarely been applied to parasite plant research and focus mainly on secondary metabolites (e.g., [1,4,17]).

The main goal of this study is to gain more knowledge about the metabolism of *C. campestris* using primary metabolic profiling and to examine how its metabolic profiling is affected by its hosts. To this end, we collected three main organs (haustoria, stem and flowers) from parasitic plants that grew on three different hosts.

## 2. Results

### 2.1. Primary Metabolic Profiles Analysis Using Gas Chromatography-Mass Spectrometry (GC-MS) Reveals a Distinct Metabolic Profile in Haustoria, Stem and Flowers with Respects to the Hosts

**Figure 1:**
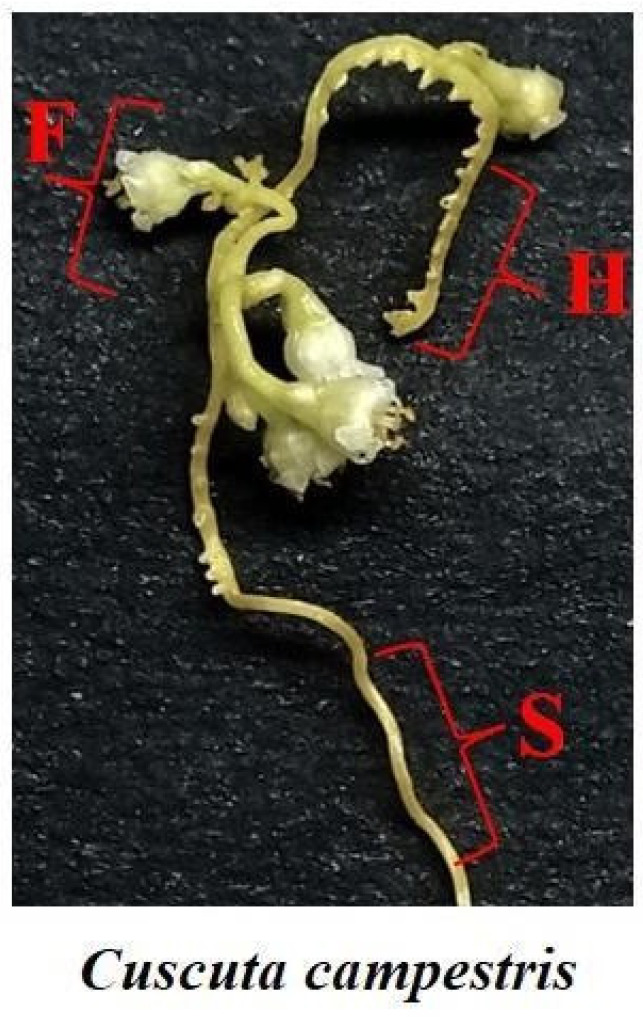
The organs of *Cuscuta campestris*, where F, H and S indicate flowers, haustoria and stem, respectively.

GC-MS analysis was performed to reveal the levels of primary metabolites in three organs of C. campestris that developed on three different hosts: *Heliotropium hirsutissimum, Polygonum equisetiforme* and *Amaranthus viridis*. These hosts come from three families: Boraginaceae, Polygonaceae and Amaranthaceae, respectively. From each of these hosts three organs, haustoria, stem and flowers (Figure 1), were collected. All the samples were collected on the same day in plants that were grown in the same wild field. The analysis enabled us to detect 59 annotated primary metabolites belonging to seven distinct biochemical groups: amino acids (15), polyols (5), tricarboxylic acid cycle (TCA) intermediates (4), organic acids (10), sugar acids (4), fatty acids (4), others (3) and sugars (14). The sugars also included three unannotated sugars (NA1, NA2 and NA3), which the GC-MS identified as sugars but could not indicate the right annotation according to the m/z ratio. The other annotated sugars and metabolites were identified by standards or by the retention index relative to alkane’s standards.

To obtain more information, the levels of these metabolites in the three organs in each parasite that developed on the three hosts were organized in Table 1. The results showed that flowers from the three hosts have relatively higher levels of the branch chain amino acids, isoleucine and valine, as well as alanine and phenylalanine. This suggests that these amino acids can synthesize and/or accumulate in the flowers of this parasite. High levels of other amino acids are found in one or two hosts, such as proline, which had high levels in the flowers of *H. hirsutissimum*, as well as tyrosine and tryptophan (both belonging to the aromatic amino acids), whose levels were found to be higher in *H. hirsutissimum* and *P. equisetiforme* (Table 1). Flowers also showed significantly higher levels of sugar acids (glucuronic acid, galactonic acid and gluconic acid), as well as sugars (fructose, talose, sugar NA3 and gentobiose). They also had higher levels of benzoic acid, gamma-butyric acid, phosphoric acid and glycerol compared to the two other organs that developed on the same parasite (Table 1). The rest of the metabolites showed a comparatively large variety between the parasite’s flowers that developed on the three hosts.

The haustoria from the parasite that developed on the three hosts tended to have high levels of sucrose, trehalose, sugar NA1, quinic acid, xylitol, ethanolamine, lumichrome and the amino acid, glutamine. The stems of C. campestris that grew on *H.* *hirsutissimum* and *P. equisetiforme* showed significantly higher levels of xylose, galactose, sugar NA2 and malic acid (Table 1). 

Taken together, the results indicate that the hosts significantly affect the levels of metabolites in the three organs. This effect is more pronounced in the stems and haustoria, and less in the flowers, which showed a greater number of common metabolites that arose in this organ relative to the two other organs.

**Figure 2:**
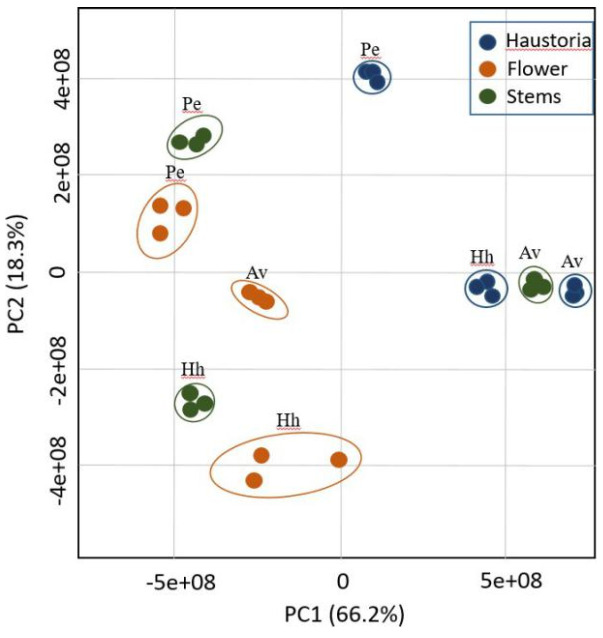
Principal component analyses (PCA) applied to *Cuscuta campestris* organs that developed on three hosts [*Heliotropium hirsutissimum* (Hh), *Polygonum equisetiforme* (Pe), *Amaranthus viridis* (Av)], according to their entire primary metabolome set of 59 metabolites. The data points are displayed as projections onto the two primary axes (eigenvectors). Variances explained by the first two components (PC1 and PC2) appear in parentheses.

The results also showed large differences in the accumulation of the metabolites between the three organs of *C. campestris* that developed on the same host. To test the general effect of the host on the accumulation of metabolites on the three different organs of *C. campestris*, the metabolic profiles of the haustoria, stem and flowers were plotted onto a principal component analysis (PCA) that mathematically quantifies the distance between variables and expresses the original data by principal components in the plotting space [18] (Figure 2). Variances were explained by the two components, PC1, which was responsible for 66.2%, and PC2, which gave a value of 18.3% of the variance. The observation that each of the organs from the three hosts, haustoria, stem and flowers, do not cluster together further strengthens the impression that each of the three hosts significantly affects the metabolic profiling of each of the three organs in different ways. The finding that the organs of *P. equisetiforme* are relatively closer to each other compared to the other two hosts suggests that this host is more affected the metabolites in the organs. The haustoria that developed on this host was relatively far from those developed on the two other hosts (Figure 2).

In addition, the results show that the host’s metabolites mainly affected the stem’s metabolites, since the distance between the clusters of this organ that developed on the three hosts was relatively far compared to the other two organs (flowers and haustoria). The clusters representing the stems, and to a lesser extent also the haustoria, were scattered on both the transverse and longitudinal axes (PC1 and PC2), while the flower components obtained from the three hosts were mainly affected by PC2 (Figure 2). This suggests that the differences between the flowers are smaller relative to the other two organs. The clusters that represent the organs developed on the parasite that infected the *A. viridis* that was influenced by PC1.

**Figure 3:**
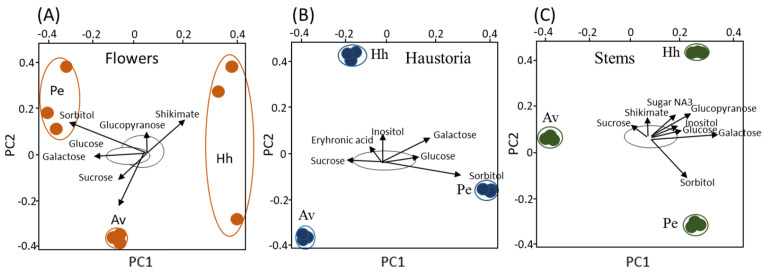
Biplot analysis applied to each of three *Cuscuta campestris* organs (haustoria, stem and flowers) that developed on each of three different host plants (**A**–**C**). The area marked with a black line is the area where most of the metabolites are located. Only the metabolites that have exceeded the boundaries of the area are marked with arrows. The data represent three replicates for each organ. The host plants are *Heliotropium hirsutissimum* (Hh), *Polygonum equisetiforme* (Pe) and *Amaranthus viridis* (Av).

To gain more knowledge about the effect of the host on the metabolites that accumulate in each organ, biplot analyses were performed using an R-based software (Figure 3). When the flowers of *C. campestris* collected from the three hosts were plotted together, it can be shown that sorbitol, shikimic acid, galactose, sucrose, glucopyranose and glucose are the metabolites that mostly affected the variance (Figure 3A). The levels of sorbitol, inositol, glucose, galactose and eryhronic acid affected the haustoria (Figure 3B). The variance of the stems was affected by sorbitol, galactose, shikimic acid, glucopyranose, sucrose, glucose and sugar NA3 (Figure 3C). Taken together, sorbitol, inositol, galactose and glucose are the main metabolites that contribute to variance when each of the organs was examined separately (Figure 3).

**Figure 4:**
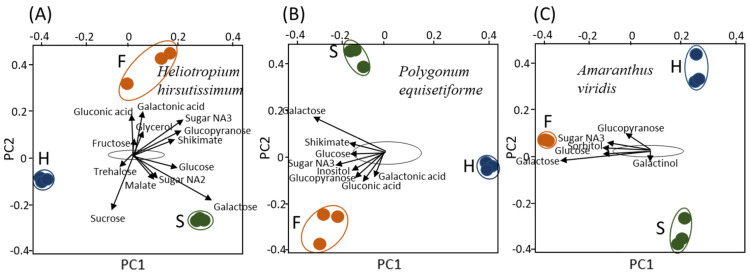
Biplot analysis applied to each of the three organs (haustoria, stem and flowers) of *Cuscuta campestris* that developed on the same host (**A**–**C**). The area marked with a black line is the area where most of the metabolites are located. Only the metabolites that have exceeded the boundaries of the area are marked with arrows. The data represent three replicates for each organ. The host plants are *Heliotropium hirsutissimum*, *Polygonum equisetiforme* and *Amaranthus viridis*; F, H and S indicate flowers, haustoria and stem, respectively.

We also performed a biplot analysis on the three organs of *C*. *campestris* that developed on each of the hosts. When the organs were collected from the parasite grown on the *H. hirsutissimum* plot together, it was shown that metabolites such as gluconic acid, glycerol, glucopyranose, sugar NA3, galactonic acid, fructose and shikimate contributed mostly to the variance of the flowers, while those contributing to the variance of the stems were galactose, glucose, sugar NA2 and malic acid (Figure 4A). Sucrose and trehalose contributed to the haustoria. The results showed that the metabolites that contributed to the variance were distributed in both PC1 and PC2. When the same analysis was made for the parasite that developed on *P*. *equisetiforme*, it was defined that as detected in the flowers of *H. hirsutissimum*, gluconic acid, glucopyranose, sugar NA3 and galactonic acid contributed to the flowers in addition to inositol. The levels of galactose, glucose and shikimate mainly contribute to the variance of the stems that developed on this host (Figure 4B). The metabolites that mostly affected the variance of the flowers of *C. campestris* collected on *A. viridis* were galactose, glucose, inositol, sugar NA3 and glucopyranose (Figure 4C). Galactinol contributes to the variance of the stems. Together, the metabolites on this host mostly contribute to PC1, while those of *H. hirsutissimum* contribute to PC1 and PC2, and those of *P. equisetiforme* were in an intermediate state between the two other hosts (A–C). Most of the metabolites in the parasites that developed on the three hosts contributed to the variance of the flowers and stems, but much less to the haustoria (Figure 4A–C).

**Figure 5:**
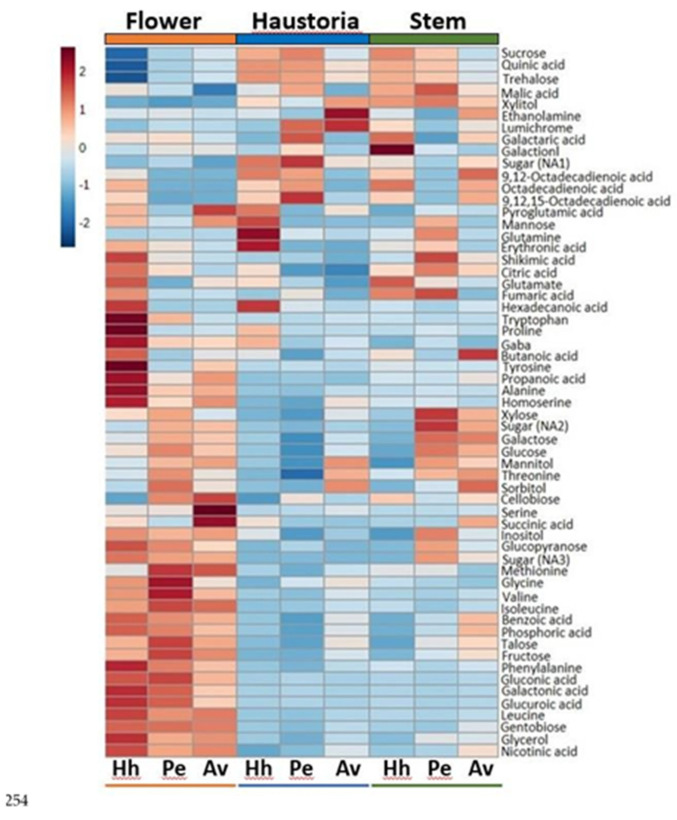
Heat-map of these 59 primary metabolites detected by GC-MS. The data represent three replicates. The host plants are *Heliotropium hirsutissimum* (Hh), *Polygonum equisetiforme* (Pe) and *Amaranthus viridis* (Av).

To further verify the effect of the host on the accumulation of metabolites in the three organs and to determine general trends, a heat-map analysis was performed (Figure 5). As indicated in the PCA analysis, the results show that the metabolic profile of the flowers that were collected from the parasite that grew on the different hosts is relatively similar (Figure 5). Compared to the stem and haustoria, the flowers accumulate high levels of several metabolites, which are comprised of several amino acids (serine, valine, isoleucine, leucine, methionine, glycine, phenylalanine, gamma-butyric acid, alanine, homoserine), sugars (glucopyranose, talose, fructose, gentobiose, sugars NA3), sugar acids (gluconic acid, galactorinc acid, glucuronic acid), the two polyols (inositol, glycerol), as well as succinic acid, propanoic acid and nicotinic acid (Figure 5). The stems and haustoria, however, tend to have relatively high levels of other metabolites compared to the flowers such as other sugars (sucrose, trehalose, sugar NA1), organic acids (quinic acid, malic acid) and polyols (xylitol).

### 2.2. Determination of Total Soluble Protein and Total Phenolic Compounds 

**Figure 6:**
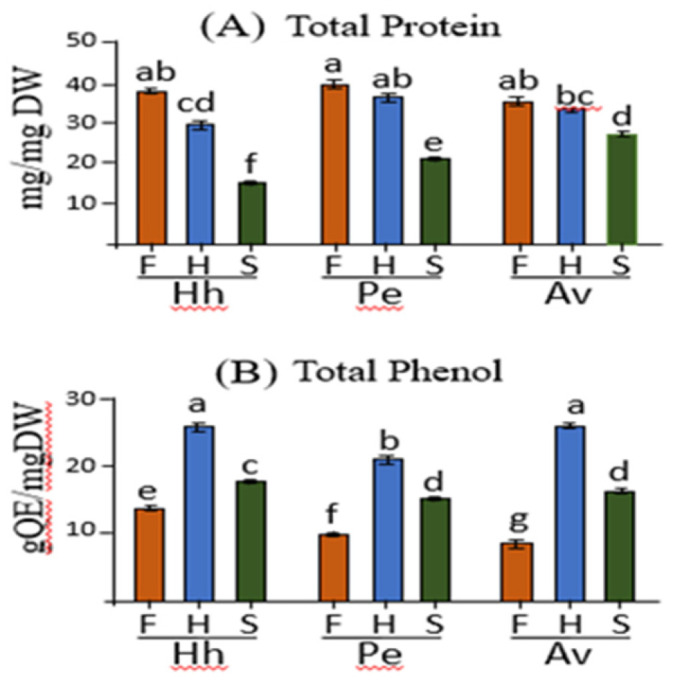
The total protein (**A**) and total phenol content (**B**) in the three organs of *Cuscuta campestris*, flowers (F), haustoria (H) and stem (S), that developed on the three host plants, *Heliotropium hirsutissimum* (Hh), *Polygonum equisetiforme* (Pe) and *Amaranthus viridis* (Av). (**A**) The total protein contents in the albumin fraction as measured using the Bradford assay; (**B**) The total phenol contents represented as mg quercetin equivalents (QE) per mg of dry weight (DW). All data shown are means ± SE of three replicates for each organ. The significance was calculated according to the Tukey-Kramer HSD test (*p* < 0.05) and is identified by different small letters.

The higher levels of most of the free amino acids in the flowers irrespective of the hosts suggested that the flowers have higher levels of proteins. To examine this assumption, a Bradford analysis was performed on the soluble protein fractions that were extracted from the different organs. The analyses revealed that the protein content indeed tends to be highest in the flowers, followed by the haustoria, whereas in the stem it is significantly lowered irrespective of the host plants (Figure 6A). In addition, the relatively high levels of the aromatic amino acids in the organs of *C. campestris* suggest that they can influence the synthesis of phenolic compounds. To verify this assumption, the levels of total soluble phenolic compounds were examined in the organs. The results demonstrated that the total phenolic content was highest in the haustoria obtained from *C. campestris* that grew on the three hosts, followed by the stem, and the lowest amount accumulated was detected in the flower (Figure 6B).

## 3. Discussion

The main aim of the current study was to determine the effect of three different hosts on the primary metabolic profile of three main organs of *C. campestris* (haustoria, stem and flowers). The leading assumption was that *C. campestris*, similar to all other holoparasite plants such as different species of *Phelipanche*, *Orobanchae*, *Conopholis* and *Epifagus* [4], relies on their hosts for carbohydrates, minerals and water to complete their life cycle [4,17,19]. Still, there is little knowledge about the question of how much of the *C. campestris*’ metabolism relies on its hosts for the other primary metabolites. We assume that if the parasite takes mostly carbohydrates from its hosts and relies on its genes to control the synthesis and accumulation of other metabolites, its metabolic profiling in each of the three organs would be relatively similar when it grows on different hosts. However, if its metabolic profiling relies mainly on its hosts’ metabolites, its metabolic profiling would differ significantly. 

The results of this study showed that the organs of *C. campestris* that developed on the three hosts have different levels of primary metabolites (Figure 2, Figure 3 and Figure 4; Table 1). This suggests that the host significantly affects the metabolic profiling of *C. campestris* and the parasite strongly relies on the host’s metabolites. The results suggest that in addition to photosynthesis products, the parasite absorbs many other primary metabolites, some of which accumulated and others were catabolized to other metabolites. 

A similar assumption that the host significantly affects metabolites levels in the parasite was also proposed for another holoparasite, *Phelipanche aegyptiaca* [8]. The hosts were also found to affect the morphological parameters of *C. campestris* that developed on three different hosts since the dry weight, size of stem length and the number of attachment sites of the parasite differed significantly between the three hosts [20]. Despite these studies, dependence on the host for different metabolites might be less necessary at the beginning of the establishment of the parasite; at least one study examined the levels of primary metabolites using GC-MS in *C. japonica* seedlings (7-day-old), and after eight days of parasitization on *Momordica charantia* as a host. It was shown that the levels of only laminaribiose (disaccharide of glucose) increased significantly in the parasite, while the levels of the other detected metabolites were not significantly altered [5]. 

As shown in the PCA and heatmap analyses, the stems were significantly affected by the host, the haustoria were less affected and the flowers the reproductive organ had relatively the most conserved profile. A similar observation was also derived from the analysis of five different organs of *P. aegyptiaca* that collected in the same mature stage, showing significant differences in the metabolites’ levels between the vegetative and reproductive organs [21]. The main metabolites that accumulated in flowers were the soluble amino acids and sugar acids (except for galactaric acid) (Table 1). The high levels of free amino acids in the flowers are in accordance with the high level of total proteins in this organ. This is similar to *P. aegyptiaca*, whose flowers have high levels of sugars, amino acids and total proteins compared to the other organs [21]. The high level of metabolites in the flowers might affect the levels of metabolites in the seeds. However, it was previously shown in *C. campestris* that the seeds had lower levels of carbohydrates and proteins compared to the stems [17]. The high levels of aromatic amino acids in flowers do not link with the observation that this organ has the lowest total phenol content. This finding is in contrast with the positive link between aromatic amino acids and total phenols in the flowers and flower buds of *P. aegyptiaca* [21]. Even though the finding that the *C. campestris*’ flowers have relatively low content of total phenols (Figure 5), measuring total phenols in the seeds and shoots of this parasitic plants in another study showed that the levels in seeds are significantly higher than in shoots, which was stable in two dodders irrespective of their different hosts [22], suggesting that seeds can produce or accumulates phenols.

Similar to flowers that show accumulations of certain metabolites, haustoria from all hosts had the highest levels of total phenols compared with the stem and flowers (Figure 5). Since the levels of the aromatic amino acids were relatively low in this organ (Table 1), it raises the possibility that the phenols were derived from the hosts. It is well known that damaged plant tissue accumulates phenols as part of the defense response [23]. Indeed, a previous report suggested that the parasitism of *C. campestris* on host plants induces the synthesis of phenolic compounds in the host [22,24,25]. The finding that the level of phenols in the haustoria differs between plants grown on different hosts also suggests that the phenols come from the host. However, to further verify this assumption, measurements of the levels of phenols in the hosts and flux analysis should be performed. 

Do the parasitic plants transport only the metabolites required for their growth? The answer is yet unknown, but it was previously suggested that *C. campestris* and *C. japonica* had no selective absorption of specific compounds from the host [17,26]. Furthermore, an additional study detected secondary metabolites in *C. reflexa* grown on two different plant species, showing that specific compounds that are synthesized in each host were present in the parasite [27]. In any case, these secondary metabolites reflect some of the metabolites that synthesized in their hosts [27]. Moreover, detecting different flavonoids in *C. reflexa* plants growing on five different hosts showed significant differences in the contents of these flavonoids, which reflected their hosts [28]. This variation in phytochemicals present in *C. reflexa* confirms that chemical constituents of the parasite depend on the nature of host and that no selection in the transformed compounds occurs. Yet, it has been suggested that some metabolites transported to dodders could be further metabolized, and indeed studies have shown that in addition to metabolites belonging to the host plant, others were metabolized by Cuscuta [19,29].

Overall, the results of this study have shown that: (i) the levels of primary metabolites of *C. campestris*’ organs were affected by their hosts; and (ii) the metabolic profile of at least the flowers is also dictated by the needs of this organ. This latter point is supported by the observation that flowers and also slightly the stems and haustoria have some metabolites that characterized their metabolic profiling, independent of the host. Moreover, the finding that the three organs that developed on the same host showed different profiles suggests that each of the organs has the ability to alter its metabolic profiling by expressing specific sets of genes. 

This metabolic study is a first step in understanding the ability of the parasite to accumulate and/or produce its own metabolites. Future studies should focus on assaying the gene expression profile of the parasite and study the enzyme activities, or other proteins involved in the synthesis and accumulation of these metabolites, in order to reveal the biochemical pathways and their regulation in the parasite. Feeding analyses using radiolabeled compounds are also required to reveal the flux from the host towards the parasite, as well as metabolic profiles analyses of the hosts.

## 4. Materials and Methods

### 4.1. Plant Material and Sample Collection

Three different organs, haustoria, stem and flowers of C. campestris plant, were collected from three hosts, *Heliotropium hirsutissimum*, *Polygonum equisetiforme* and *Amaranthus viridis*. All the organs were collected on the same day from the same wild field in Kibbutz Dan (northern Israel) (Figure 1). The parasite samples were separated, frozen in liquid nitrogen and lyophilized. The lyophilized organs were then ground to fine powder by mortar and pestle. 

### 4.2. Extraction and Analysis of Primary Metabolites Using GC-MS 

Primary metabolites were extracted from 20 mg dried weight of the haustoria, stem and flowers. The samples were homogenized using a Restch MM 301 homogenizer in 1000 µL of methanol/chloroform/double distill water (DDW) (2.5:1:1) at 4 °C. Norleucine (4.6 µL of 2 mg per ml) was added as an internal standard. After short vortex and 10 min of centrifugation at 20,000 g at 4 °C, 1,000 µL the supernatant was collected to a new tube. The lower phase kept for fatty acid analysis. In this case, 300 µL distil DDW and 300 µL chloroform were added. The samples were vortexed for 1 min and settled for 5 min at room temperature, followed by 10 min of centrifugation at 20,000 g at 4 °C. In this case, 300 µL from the upper phase were dried using speadvac. The dried samples were then dissolved and treated for 2 h at 37 °C with 40 µL 20 mg/mL methoxyamine hydrochloride in pyridine, followed by derivatization for 30 min in N-methyl-N(trimethylsilyl)-trifluoro-acetamide at 37 °C with vigorous shaking. Sample volumes of 1 µL were injected into a GC- 414 MS system. The single-ion mass method was used for soluble amino acid determination with the BP5MS capillary column (SGE-gc; 30 m, 0.25 mm i.d. and 0.25 mm thickness), while the total-ion-count method was used for metabolic profiling and separation using the VF-5ms capillary column (Agilent; 30 m + 10 m EZ-guard, 0.25 mm i.d. and 0.25 mm thicknesses). All analyses were carried out on a GC-MS system (Agilent 7890A) coupled with a mass selective detector (Agilent 5975c) and a Gerstel multipurpose sampler MPS2 [30]. Peak finding, peak integration and retention time correction were performed using the Agilent GC/MSD Productivity ChemStation package (www.agilent.com, last accessed on 25 September 2021). Peak areas were normalized to integral standard (norleucine) signal. For amino acids analysis, amino acid standards of 200, 100, 50, 25 and 5 µM were injected to establish quantification curves, and the amounts of amino acids were calculated accordingly [21].

For fatty acids determination, 200 µL of the lower phase containing fatty acid were then transferred to a new tube and dried with nitrogen gas, followed by the addition of 300 µL of methanol with 2% H2SO4. After vortexed the tubes incubated at 85 °C for one hour under shaking conditions. Then, 300 µL of DDW and 300 µL hexane were added, the blend was mixed well and centrifuged for 20 min at 20,000g. Approximately 150 µL aqueous phase was transferred to the GC-MS tube and analyzed by GC-MS. Heptadecanoic acid was used as an internal standard. 

The annotations of the metabolites were made using standards or the retention index relative to alkanes standards. The corresponding mass spectra and retention time indices were compared with standard substances and commercially available electron mass spectrum libraries available from the National Institute of Standards and Technology and Max Planck Institute for Plant Physiology, Golm, Germany (http://www.mpimpgolm. mpg.de/mms-library/, last accessed on 25 September 2021).

### 4.3. Total Soluble Protein Determination

For total soluble protein determination, 5 mg dried weight of the haustoria, stem and flowers were grounded in 200 µL buffer phosphate pH=7.8 with a protease inhibitor cocktail (Sigma Aldrich). After two centrifugation cycles (20,800 g for 5 min), total protein was determined using a Bradford reagent (Bio-Rad) in three sample concentrations. Bovine serum albumin was used as a standard. Total phenolic compounds content determination For total phenolic compounds content determination, 5 mg dried weight of the haustoria, stem and flowers were ground in 0.5 mL DDW, the colorimetric method that modified the Ben Nasr method for small volumes [31] was used. Six µL of the extraction sample was loaded on a 96 well ELISA plate. To each well, 50 µL of 10% Folin-Ciocalteu reagent and 40 μL of 7.5% (*w/v*) Na_2_CO_3_ were added. The plate was incubated for 40 min at 37 °C and then read at 765 nm. A standard curve was created using quercetin.

### 4.4. Statistical Analyses 

For the metabolites study, three biological replicate samples were taken of each organ (haustoria, stem and flowers). The data represent the mean of three independent replications for the metabolites and five for the phenol and total protein. Statistical significance was evaluated using JMP software version 8.0 (SAS Institute Inc., Cary, NC). Significant differences between treatments were calculated according to the Tukey-Kramer HSD test (p < 0.05). Principal component analysis (PCA) and a heatmap of GC-MS data were con- ducted using the MetaboAnalyst 3.0 comprehensive tool (http://metaboanalyst.ca/, last accessed on 25 September 2021; [18] with auto scaling (mean-centered and divided by the standard deviation of each variable) manipulations. Graphs were compiled using GraphPad Prism 5.01 scientific software (http://www. graphpad.com, last accessed on 25 September 2021).

## Figures and Tables

**Table 1:** The levels of individual primary metabolites in the organs (flowers, haustoria and stem) of *Cuscuta campestris* that developed on three hosts, *Heliotropium hirsutissimum*, *Polygonum equisetiforme* and *Amaranthus viridis* as detected by using GC-MS. The data represent the mean ± SE of three biological replicates from each organ. Values are relative peak areas normalized to the norleucine internal standard. Quantities of soluble amino acids were calculated as nmol/g dry weight. Orange, blue and green colors correspond to metabolites that detected in flower, haustoria and stem. Significance was calculated according to the Tukey Kramer HSD test (*p* < 0.05) and identified by letters. NA, non-annotated sugars; ND, not detected or the values were less than 1.

	Flowers			Haustoria			Stem		
Metabolites	Hh	Pe	Av	Hh	Pe	Av	Hh	Pe	Av
**Sugars**									
**Sucrose**	229 ± 46 ^e^	366 ± 16 ^cd^	2 ± 0.03 ^f^	710 ± 9 ^a^	463 ± 17 ^bc^	763 ± 40 ^a^	535 ± 8 ^b^	463 ± 10 ^bc^	284 ± 1 ^de^
**Glucose**	373 ± 36 ^bc^	473 ± 21 ^ab^	325 ± 5 ^bc^	183 ± 9 ^bc^	253 ± 7 ^bc^	30 ± 1 ^c^	747 ± 215 ^a^	485 ± 17 ^ab^	32 ± 2 ^c^
**Trehalose**	38 ± 8 ^e^	78 ± 2 ^c^	372 ± 6 ^d^	159 ± 2 ^a^	110 ± 4 ^b^	152 ± 7 ^a^	113 ± 1 ^b^	107 ± 1 ^c^	55 ± 1 ^de^
**Galactose**	828 ± 69 ^c^	988 ± 36 ^b^	794 ± 4 ^cd^	614 ± 18 ^e^	659 ± 16 ^de^	284 ± 12 ^f^	1206 ± 12 ^a^	1318 ± 30 ^a^	168 ± 5 ^f^
**Talose**	54 ± 2 ^b^	65 ± 1 ^a^	41 ± 0.2 ^c^	10 ± 1 ^e^	32 ± 1 ^d^	3 ± 0.2 ^f^	27 ± 1 ^d^	42 ± 1 ^c^	1 ± 0.07 ^f^
**Fructose**	115 ± 5 ^ab^	137 ± 4 ^a^	97 ± 1 ^bc^	14 ± 1 ^e^	54 ± 1 ^d^	11 ± 0.5 ^e^	48 ± 10 ^d^	81 ± 9 ^c^	5 ± 0.5 ^e^
**Glucopyranose**	367 ± 14 ^a^	255 ± 54 ^ab^	18 ± 0.9 ^d^	24 ± 1 ^d^	18 ± 0.8 ^d^	76 ± 3 ^d^	238 ± 38 ^bc^	122 ± 19 ^cd^	9 ± 0.5 ^d^
**Mannose**	2 ± 0.1 ^bc^	1 ± 0.02 ^bc^	460 ± 2 ^a^	4 ± 0 ^b^	ND	ND	2 ± 0.02 ^bc^	ND	ND
**Cellobiose**	3 ± 1 ^c^	39 ± 2 ^a^	7 ± 0.4 ^c^	1 ± 0.1 ^c^	13 ± 1 ^c^	31 ± 3 ^ab^	16 ± 7 ^bc^	30 ± 2 ^ab^	13 ± 0.9 ^c^
**Gentobiose**	11 ± 0.5 ^a^	8 ± 1 ^b^	2 ± 0.1 ^de^	1 ± 0.06 ^de^	2 ± 0.01 ^d^	1 ± 0.02 ^e^	1 ± 0.02 ^de^	4 ± 0.6 ^c^	1 ± 0.05 ^e^
**Xylose**	11 ± 1 ^abc^	11 ± 1 ^ab^	5 ± 0.3 ^cd^	3 ± 0.09 ^de^	7 ± 0.4 ^bcd^	1 ± 0.01 ^e^	16 ± 2 ^a^	13 ± 1 ^a^	1 ± 0.02 ^e^
**Sugar (NA1)**	1 ± 0.04 ^c^	2 ± 0.3 ^c^	ND	5 ± 3 ^a^	2 ± 0.05 ^ab^	6 ± 0.9 ^a^	1 ± 0.01 ^c^	2 ± 0.2 ^ab^	1 ± 0.01 ^c^
**Sugar (NA2)**	50 ± 1 ^c^	123 ± 2 ^b^	98 ± 2 ^b^	23 ± 1 ^cd^	32 ± 0.8 ^cd^	6 ± 0.6 ^d^	193 ± ^a^	134 ± 2 ^b^	4 ± 0.8 ^d^
**Sugar (NA3)**	513 ± 4 ^a^	358 ± 9 ^b^	297 ± 3 ^b^	5 ± 0.7 ^d^	2 ± 0.01 ^d^	13 ± 0.8 ^d^	379 ± 6 ^ab^	232 ± 23 ^c^	1 ± 0.07 ^d^
**Sugar acids**									
**Galactonic acid**	119 ± 1.9 ^b^	83 ± 1 ^c^	148 ± 4 ^a^	1 ± 0.02 ^d^	2 ± 0.1 ^d^	5 ± 0.4 ^d^	2 ± 0.4 ^d^	7 ± 0.1 ^d^	2 ± 0.06 ^d^
**Glucuronte**	15 ± 0.9 ^a^	11 ± 0.5 ^b^	109 ± 1 ^c^	ND	ND	1 ± 0.02 ^d^	ND	1 ± 0.04 ^d^	ND
**Gluconic acid**	242 ± 38 ^a^	207 ± 5 ^a^	40 ± 1 ^b^	2 ± 0.01 ^b^	ND	16 ± 1 ^b^	4 ± 0.6 ^b^	18 ± 1 ^b^	5 ± 0.5 ^b^
**Galactaric acid**	4 ± 0.4 ^cd^	3 ± 0.4 ^d^	5 ± 0.8 ^b^	2 ± 0.06 ^e^	2 ± 0.06 ^e^	6 ± 0.7 ^a^	1 ± 0.05 ^e^	4 ± 0.9 ^bc^	2 ± 0.3 ^e^
**TCA metabolites**									
**Malic acid**	329 ± 10 ^b^	244 ± 5 ^c^	150 ± 1 ^d^	332 ± 5 ^b^	226 ± 5 ^c^	381 ± 8 ^a^	365 ± 15 ^ab^	328 ± 3 ^b^	150 ± 1 ^d^
**Citric acid**	264 ± 13 ^a^	191 ± 3 ^cd^	152 ± 1 ^f^	241 ± 2 ^ab^	161 ± 2 ^ef^	186 ± 5 ^de^	219 ± 7 ^bc^	230 ± 2 ^b^	90 ± 0.9 ^g^
**Succinic acid**	44 ± 2 ^abc^	19 ± 0.7 ^bc^	77 ± 1 ^a^	43 ± 15 ^abc^	11 ± 0.8 ^c^	13 ± 0.6 ^c^	15 ± 1 ^c^	61 ± 21 ^ab^	6 ± 0.4 ^c^
**Fumaric acid**	73 ± 2 ^a^	22 ± 0.7 ^cd^	21 ± 0.9 ^d^	17 ± 0.9 ^de^	8 ± 0.5 ^ef^	40 ± 0.4 ^b^	76 ± 4 ^a^	13 ± 0.3 ^f^	30 ± 1 ^c^
**Organic acids**									
**Shikimic acid**	645 ± 45 ^a^	309 ± 32 ^bc^	224 ± 3 ^cd^	279 ± 13 ^bc^	173 ± 9 ^de^	278 ± 16 ^bc^	548 ± 17 ^a^	380 ± 12 ^b^	123 ± 1 ^e^
**Benzoic acid**	16 ± 0.8 ^a^	12 ± 0.7 ^b^	9 ± 0.9 ^c^	5 ± 0.4 ^e^	8 ± 0.6 ^d^	3 ± 0.5 ^f^	6 ± 0.6 ^e^	12 ± 0.5 ^b^	1 ± 0.1 ^g^
**Pyroglutamate**	10 ± 0.7 ^ab^	5 ± 0.6 ^de^	9 ± 0.6 ^ab^	12 ± 1 ^a^	7 ± 0.5 ^bcd^	5 ± 0.4 ^d^	6 ± 0.5 ^cd^	6 ± 1 ^bcd^	1 ± 0.04 ^e^
**Nicotinic acid**	3 ± 0.2 ^a^	2 ± 0.02 ^ab^	2 ± 0.3 ^ab^	1 ± 0 ^bc^	1 ± 0.01 ^abc^	1 ± 0.01 ^bc^	1 ± 0.06 ^bc^	2 ± 0.02 ^ab^	1 ± 0.06 ^c^
**Quinic acid**	1 ± 0.01 ^d^	1 ± 0.1 ^cd^	42 ± 1 ^cd^	4 ± 0 ^a^	2 ± 0.3 ^bcd^	4 ± 0.1 ^a^	2 ± 0.2 ^bc^	2 ± 0.3 ^bcd^	1 ± 0.03 ^cd^
**Butanoic acid**	1 ± 0.2 ^a^	ND	ND	ND	ND	ND	ND	1 ± 0.01 ^a^	ND
**Propanoic acid**	23 ± 1 ^a^	10 ± 0.3 ^bcd^	12 ± 0.4 ^b^	6 ± 0.5 ^def^	5 ± 0.6 ^ef^	7 ± 0.6 ^de^	7 ± 1 ^cde^	11 ± 1 ^bc^	3 ± 0.02 ^f^
**Phosphoric acid**	327 ± 4 ^a^	246 ± 1 ^b^	174 ± 3 ^c^	99 ± 6 ^e^	147 ± 5 ^d^	49 ± 0.4 ^f^	110 ± 7 ^e^	241 ± 4 ^b^	25 ± 0.4 ^g^
**Erythronic acid**	86 ± 6 ^b^	51 ± 1 ^c^	32 ± 0.6 ^d^	142 ± 6 ^a^	148 ± 1 ^e^	19 ± 0.9 ^de^	65 ± 2 ^c^	32 ± 1 ^d^	23 ± 1 ^de^
**GABA**	57 ± 1 ^a^	22 ± 0.2 ^c^	18 ± 0.9 ^c^	34 ± 1 ^b^	13 ± 0.7 ^d^	6 ± 0.7 ^e^	10 ± 0.9 ^d^	2 ± 0.4 ^f^	1 ± 0.07 ^f^
**Polyols**									
**Mannitol**	87 ± 35 ^bc^	135 ± 2 ^ab^	134 ± 1 ^ab^	53 ± 2 ^cd^	173 ± 4 ^a^	3 ± 0.8 ^d^	156 ± 4 ^a^	139 ± 2 ^ab^	4 ± 2 ^d^
**Xylitol**	26 ± 11 ^cd^	33 ± 1 ^abcd^	30 ± 0.1 ^b^	50 ± 1 ^a^	46 ± 0.9 ^abc^	45 ± 1 ^abc^	43 ± 1 ^abc^	47 ± 2 ^ab^	18 ± 1 ^d^
**Inositol**	646 ± 24 ^a^	488 ± 12 ^c^	2 ± 0 ^g^	470 ± 5 ^cd^	317 ± 5 ^e^	260 ± 14 ^e^	563 ± 10 ^b^	430 ± 9 ^d^	96 ± 3 ^f^
**Galactinol**	20 ± 2 ^c^	33 ± 2 ^bc^	76 ± 1 ^ab^	13 ± 1 ^c^	7 ± 0.4 ^c^	61 ± 24 ^ab^	24 ± 1 ^bc^	7 ± 0.6 ^c^	63 ± 1 ^a^
**Sorbitol**	187 ± 80 ^cd^	686 ± 11 ^ab^	303 ± 2 ^c^	75 ± 3 ^de^	677 ± 16 ^b^	10 ± 1 ^e^	229 ± 5 ^e^	818 ± 9 ^a^	13 ± 1 ^e^
**Others**									
**Ethanolamine**	83 ± 8 ^cd^	70 ± 1 ^cd^	56 ± 0 ^def^	88 ± 2 ^c^	157 ± 4 ^a^	63 ± 3 ^cde^	41 ± 6 ^ef^	122 ± 12 ^b^	29 ± 1 ^f^
**Glycerol**	265 ± 1 ^a^	148 ± 2 ^b^	146 ± 2 ^b^	54 ± 2 ^e^	69 ± 2 ^de^	22 ± 0.9 ^f^	88 ± 3 ^cd^	96 ± 13 ^c^	15 ± 1 ^f^
**Lumichrome**	ND	1 ± 0.07 ^bc^	ND	ND	2 ± 0.1 ^a^	2 ± 0.03 ^a^	ND	1 ± 0.05^b^	ND
**Fatty acids**									
**Hexadecanoate**	10 ± 1 ^a^	ND	1 ± 0.1 ^bc^	10 ± 0.07 ^a^	1 ± 0.01 ^c^	8 ± 0.8 ^a^	1 ± 0.02 ^c^	7 ± 3 ^a^	7 ± 0.3 ^ab^
**Octadecanoate**	2 ± 1 ^a^	ND	ND	1 ± 0.7 ^a^	ND	2 ± 0.07 ^a^	ND	2 ± 1 ^a^	2 ± 0.01 ^a^
**Octadecadienoate**	3 ± 1 ^c^	ND	ND	7 ± 0.08 ^ab^	1 ± 0.01 ^d^	6 ± 0.7 ^ab^	1 ± 0.01 ^d^	7 ± 0.1 ^a^	5 ± 0.7 ^bc^
**Octadecatrienoat**	6 ± 0.5 ^ab^	ND	1 ± 0.03^b^	7 ± 2 ^ab^	2 ± 2 ^b^	12 ± 0.8 ^a^	1 ± 1 ^b^	8 ± 3 ^ab^	7 ± 0.7 ^ab^
**Amino acids**									
**Alanine**	1210 ± 44 ^a^	888 ± 18 ^b^	965 ± 74 ^b^	521 ± 87 ^c^	665 ± 30 ^c^	516 ± 3 ^c^	695 ± 24 ^c^	665 ± 39 ^c^	655 ± 15 ^c^
**Valine**	3782 ± 232 ^b^	5324 ± 205 ^a^	3347 ± 48 ^b^	1124 ± 47 ^d^	1927 ± 81 ^c^	851 ± 72 ^d^	1183 ± 35 ^cd^	1601 ± 120 ^cd^	1307 ± 95 ^cd^
**Serine**	5200 ± 18 8^b^	5401 ± 107 ^b^	9591 ± 1504 ^a^	2890 ± 173 ^c^	4495 ± 265 ^bc^	3031 ± 178 ^bc^	3194 ± 71 ^bc^	3704 ± 722 ^bc^	2893 ± 231 ^bc^
**Leucine**	2414 ± 70 ^a^	2113 ± 26 ^abc^	2180 ± 103 ^ab^	768 ± 12 ^abc^	1037 ± 6 ^cd^	659 ± 36 ^d^	973 ± 9 ^d^	1130 ± 19b ^cd^	1055 ± 39 ^cd^
**Threonine**	627 ± 19 ^ab^	737 ± 12 ^a^	590 ± 167 ^ab^	551 ± 8 ^b^	713 ± 27 ^ab^	439 ± 24 ^b^	707 ± 5 ^ab^	729 ± 40 ^a^	621 ± 24 ^ab^
**Isoleucine**	1199 ± 37 ^a^	1374 ± 24 ^a^	1290 ± 57 ^a^	566 ± 10 ^bc^	794 ± 13 ^b^	600 ± 29 ^bc^	601 ± 12 ^c^	751 ± 18 ^bc^	767 ± 31 ^b^
**Proline**	1026 ± 58 ^a^	198 ± 15 ^cd^	279 ± 22 ^cd^	639 ± 13 ^b^	177 ± 41 ^cd^	127 ± 18 ^d^	328 ± 35 ^c^	153 ± 49 ^cd^	179 ± 11 ^cd^
**Glycine**	782 ± 53 ^a^	921 ± 166 ^a^	637 ± 74 ^a^	311 ± 47 ^a^	614 ± 38 ^a^	544 ± 30 ^a^	440 ± 81 ^a^	369 ± 80 ^a^	510 ± 40 ^a^
**Homoserine**	327 ± 21 ^a^	252 ± 8 ^abc^	267 ± 28 ^abcd^	176 ± 2 ^cd^	238 ± 29 ^ab^	158 ± 14 ^d^	223 ± 1 ^abcd^	204 ± 4 ^bcd^	221 ± 13 ^abcd^
**Methionine**	270 ± 9 ^ab^	396 ± 4 ^a^	367 ± 19 ^a^	215 ± 21 ^b^	234 ± 47 ^b^	162 ± 5 ^b^	267 ± 5 ^ab^	197 ± 13 ^b^	270 ± 7 ^ab^
**Phenylalanine**	562 ± 16 ^a^	499 ± 4 ^ab^	429 ± 26 ^abc^	241 ± 1 ^bcd^	300 ± 2 ^cd^	193 ± 1 ^d^	281 ± 4 ^cd^	327 ± 5 ^bcd^	327 ± 18 ^bcd^
**Glutamate**	20294 ± 110 ^a^	7387 ± 243 ^cd^	14981 ± 789 ^ab^	15377 ± 295 ^bcd^	4623 ± 1194 ^d^	11007 ± 1358 ^bc^	14600 ± 865 ^abc^	12221 ± 1286 ^ab^	20197 ± 402 ^a^
**Glutamine**	2130 ± 152 ^d^	7629 ± 895 ^cd^	5743 ± 901 ^cd^	41281 ± 3025 ^a^	8196 ± 661 ^cd^	15172 ± 1118 ^b^	35770 ± 1484 ^a^	8322 ± 614 ^bc^	13122 ± 1049 ^bc^
**Tyrosine**	26612 ± 101 ^a^	2400 ± 197 ^b^	15255 ± 181 ^ab^	3395 ± 463 ^ab^	1318 ± 84 ^b^	5891 ± 573 ^ab^	7072 ± 539 ^ab^	7025 ± 224 ^ab^	9318 ± 943 ^ab^
**Tryptophan**	4109 ± 113 ^a^	2628 ± 87 ^b^	1047 ± 214 ^c^	765 ± 193 ^c^	1251 ± 27 ^c^	666 ± 70 ^c^	727 ± 78 ^c^	833 ± 124 ^c^	1086 ± 252 ^c^

## Data Availability

Not applicable.
